# Contraception and Catholicism in the Twentieth Century: Transnational Perspectives on Expert, Activist and Intimate Practices

**DOI:** 10.1017/mdh.2020.1

**Published:** 2020-04

**Authors:** Agata Ignaciuk, Laura Kelly

**Affiliations:** 1 Institute of Ethnology and Cultural Anthropology, Faculty of History, University of Warsaw, Poland Email: ignaciuk.agata@gmail.com; 2 School of Humanities, University of Strathclyde, Level 4, Lord Hope Building, 141 St James Road, Glasgow G4 0LT, UK Email: L.e.kelly@strath.ac.uk

**Keywords:** Birth Control, Catholicism, Contraception, Family planning, Activism

## Abstract

This special issue uses Catholicism as a thread to bring together five contributions to the transnational history of contraception. The articles, which cover examples from Western and East-Central Europe, East Africa and Latin America, all explore the complex interplay between users and providers of birth control in contexts marked by prevalence of the Catholic religion and/or strong political position of the Catholic Church. In the countries examined here, Brazil, Belgium, Poland, Ireland and Rwanda, Catholicism was the majority religion during the different moments of the long twentieth century the authors of this special issue focus on. Using transnationalism as a perspective to examine the social history of the entanglements between Catholicism and contraception, this special issue seeks to underscore the ways in which individuals and organisations used, adapted and contested local and transnational ideas and debate around family planning. It also examines the role of experts and activist groups in the promotion of family planning, while paying attention to national nuances in Catholic understandings of birth control. The contributions shed light on the motivations behind involvement in birth control activism and expertise, its modus operandi, networking strategies and interactions with men and women demanding contraceptive information and technology. Moreover, through the use of oral history, as well as other print sources such as women’s magazines, this collection of articles seeks to illustrate ‘ordinary’ men and women’s practices in the realm of reproductive health.

## 1 Catholicism and Contraception: A Transnational History

Over the past two decades, historical disciplines, including the history of medicine and health, have witnessed a boom of interest in perspectives going beyond and across local and national contexts. As Sanjoy Bhattacharya has noted, these perspectives have been, often interchangeably, termed as ‘global’, ‘international’ and ‘transnational’, and each of these terms can convey a number of conceptual paradigms.^1^ In a recent analysis of health policies in interwar Europe, historian Josep L. Barona has emphasised the interest in ‘entanglements, clashes, rejections, obstacles, rivalries and differences’^2^ as what characterises the transnational approach. Barona evokes Akira Iriye and Pierre-Yves Saunier’s definition of transnational history as an approach (rather than a historical theory or a research method) which deals with the circulation of bodies, commodities, ideas and patterns ‘over, across, though, beyond, above, under or in-between policies and societies’.^3^

This approach, shared by the contributors to this special issue, focuses, in concordance with what Jürgen Osterhammel has proposed, on the interrelation of local factors and ‘transfers from outside in achieving specific outcomes’, and is sensitive to borders and boundaries, exchanges, flows, and streams. Osterhammel has pointed specifically to religion, as one of the phenomena whose ‘social influence is not contained within political boundaries’.^4^ As numerous scholars, whose work we discuss below, have shown, technologies, as well as expert and activist practices linked to contraception, have also circulated across these boundaries. This circulation has been particularly intense during the long twentieth century, with the advent of fertility awareness-based methods, the contraceptive and abortion pill and the intrauterine device (IUD), which all travelled transnationally aided by increasingly organised – and international – family planning movements, both secular and Catholic.

This special issue uses Catholicism as the thread to bring together five contributions to the transnational history of contraception. Indeed, the Catholic Church is both a local and global institution at the same time and in a number of ways. The interplay between the Vatican and local hierarchies has often been complex, especially around controversial issues such as contraception, on which the position of the Holy See has remained essentially unchanged since the publication of the encyclical *Humanae Vitae* (1968), which solidified a ban on all ‘artificial’ contraceptive methods for Catholic spouses. The decades previous to the *Humanae Vitae* had been witness to diversification of positions regarding birth control and abortion within the Catholic community, beginning with the approval of the rhythm method under *Castii Connubii* in 1930. *Humanae Vitae* came at a crucial moment in terms of the history of birth control – the question of contraception had become the topic of heated debate, particularly with the advent of the contraceptive pill in Europe and America from the early 1960s. Many Catholics had hoped that the 1968 papal encyclical would mark the beginning of a more liberal attitude from the Church hierarchy in relation to the issue of family planning. However, this papal encyclical reinforced the Catholic Church’s position on birth control. The Church’s position was that only the use of the ‘safe period’ could be permitted as a form of contraception. The encyclical argued that the use of artificial birth control methods ‘could open wide the way for marital infidelity and a general lowering of moral standards’.^5^ Indeed, the Catholic Church’s stance on contraception has continued to have a global impact. While responses to the fiftieth anniversary of the encyclical in 2018 were generally muted, there were some opposing statements, including the publication of a report by pro-choice advocacy group Catholics for Choice, which emphasised the impact of the ban on global health, such as in efforts to contain HIV/AIDS in Africa.^6^ In addition, those identifying as Catholic further negotiate the norms proposed by the hierarchy, whether ‘global’ or local. The local hierarchies interact with local democratic or dictatorial governments, the media and medical professionals, which have been consolidated through the twentieth century as key providers of contraceptive advice and family planning experts.

The articles in this special issue, which cover examples from western and east-central Europe, East Africa and Latin America, all explore the complex interplay between users and providers of birth control in contexts marked by prevalence of the Catholic religion and/or strong political position of the Catholic Church. In the countries examined here – Brazil, Belgium, Poland, Ireland and Rwanda – Catholicism was the majority religion during the different moments of the long twentieth century the authors of this special issue focus on. They also underscore the ways in which individuals and organisations used, adapted and contested local and transnational ideas and debate around family planning. We argue that this relationship between users and providers remains key in defining material and symbolic access to contraception, historically and today.

Using transnationalism as a perspective to examine the social history of the entanglements between Catholicism and contraception, this special issue also seeks to broaden our understanding of the role of experts and activist groups in the promotion of family planning, while paying attention to local and national nuances in Catholic understandings of birth control. It highlights the ways in which birth control-related activism in and beyond the medical profession promoted debates about contraception and abortion laws in and beyond local contexts of Brazil, Belgium, Poland, Ireland and Rwanda, and shows how different groups fought for (or against) the right to contraception with (or with the support of) local and global Catholic hierarchies, political regimes and the international birth/population control movement. The contributions shed light on the motivations behind involvement in birth control activism and expertise, its modus operandi, networking strategies and interactions with men and women demanding contraceptive information and technology. Moreover, through the use of oral history, as well as print sources such as women’s magazines, this collection of articles seeks to illustrate ‘ordinary’ men and women’s practices in the realm of reproductive health.

This special issue builds upon, dialogues with and contributes to a number of areas in the history of contraception where transnational perspectives have been particularly strong: the history of contraceptive technologies, ‘ordinary’ people’s practices and activism/expertise.

## 2 Technologies

A significant amount of the historiography on birth control has examined the rise of new contraceptive technologies. Examinations of the history of the contraceptive pill have dominated this literature. Historians such as Lara Marks have explored the history of the contraceptive pill as a transnational contraceptive technology *par excellence*, with ideas and products circulating from Europe to Mexico, and back and forth between the United States and the pill testing sites in developing countries, but most approaches to the history of the pill are national or at best comparative.^7^ Similarly, there has been important research conducted on the contraceptive pill in the United States, chronicling its development, testing, introduction and impact, as well as medical professional and feminist debates around it.^8^ Hera Cook’s work, for instance, tracks the history of contraception in England, in particular, focusing on the introduction of the contraceptive pill in the 1960s, and how this impacted on women’s lives.^9^ Similarly, there have been numerous studies of the pill’s reception and the role of the medical profession in its introduction, and the significance of the doctor–patient relationship in European countries such as Spain, Portugal and West Germany.^10^ Less scholarship has focused on the rise of other contraceptive technologies; however, there have been important studies which have explored the history of contraceptive technologies in the era before the pill.^11^ In addition, there have been numerous interventions which have examined the history of intrauterine devices, Depo-Provera, vasectomy, laparoscopy, spermicides and the morning-after pill in certain geographical contexts, but there is evidently need for further studies of these technologies in other regions.^12^ Furthermore, recent studies by Claire Jones, Jessica Borges and Ben Mechen have expanded our understanding of contraceptive consumption in Britain.^13^ Contributing to this scholarship, the articles in this special issue show the malleable meanings of contraceptive practices (like the rhythm in Kuźma-Markowska and Ignaciuk’s article) or technologies such as female sterilisation (Roth) and the contraceptive pill (Kelly, Crosetti), and ways these meanings were shaped through both local and international policies, interventions and ideologies.

In particular, Cassia Roth’s article uses the medical and media debates about female sterilisation technologies in Brazil as a lens through which she examines entanglements between Catholicism, eugenics, the state and physicians during the late nineteenth and the first third of the twentieth century, a crucial moment for the development of medical specialities focused on treating – and scrutinising – the female body and mind. Tracing the debates around the controversial sterilisation procedure proposed and practised by the Italian physician Abel Parente in 1890, and their aftermath in the early twentieth century, Roth shows how the understanding of female sterilisation shifted between a practice that could either prevent or cause degeneration of Brazilian women. She demonstrates how these shifts were informed by transnational debates and movements around eugenics, in its positive and negative version, as well as conflicts and collaborations with the Brazilian Catholic Church, itself navigating between alignment and separation with the expanding nation-state. The medical profession, especially gynaecologists, obstetricians and psychiatrists, proposed themselves as key experts in these debates, claiming reproduction as their sphere of authority for the sake of the healthy nation reproduced from and by the female bodies doctors claimed to be preventing from degeneration.

Laura Kelly’s article explores the debates about the contraceptive pill in the later-twentieth-century Ireland. Relying on memoirs, press accounts and oral history interviews, as well as archival material, Kelly examines Irish trajectories of the pill in three realms: women’s contraceptive practices, expert discourses and activism. The pill was available on the Irish market from the mid-1960s as a therapeutic drug officially prescribed to regulate the menstrual cycle, a circumvention on the ban on contraception that was in place in the country until 1979. As in other predominantly Catholic countries with restrictions on the circulation of contraceptives, the emergence of the pill was an important catalyser for the opening of medical, media and social debates around family planning. Kelly demonstrates how, while access to the pill was stratified across the lines of class and place of residence, women from all social backgrounds navigated the legal, medical and religious norms around contraception in order to access birth control – and broader control over their reproductive lives. The author shows how the possibility of obtaining the pill was a by-product of a determined patient–doctor relationship, often informed by religious beliefs of the practitioner. She also traces the anti- and pro-contraception activism that developed in Ireland in the 1970s, unravelling unexpected itineraries of some of the arguments in the transnational debates around the pill. For instance, in its media interventions in the mid-1970s, the anti-contraception organisation Irish Family League echoed US women’s health movement arguments about the pill as generator of huge benefits for the pharmaceutical industry while having harmful side effects.

## 3 ‘Ordinary’ People’s Practices

In recent years, historians have attempted to integrate the experiences of ‘ordinary’ men and women to the history of birth control in the twentieth century. Kate Fisher and Simon Szreter’s oral history research has been particularly significant in this realm. Fisher’s 2006 study explored the experiences of men and women born between 1900 and 1930 in England and Wales about their experiences of and attitudes to family planning.^14^ Similarly, Fisher and Szreter’s 2010 study also drew on oral history testimonies in order to explore individuals’ experiences of sex, marriage and birth control in the era before the sexual revolution.^15^ Importantly, studies by Rusterholz and by Gervais and Gavreau highlight how men and women negotiated the tension between the teachings of the Catholic Church, their faith and their reproductive health choices. Other studies of the history of Catholicism in ‘ordinary’ men and women’s lives have also touched on issues surrounding sexuality and birth control.^16^ For instance, Alana Harris has argued that not only have Catholic men and women’s ‘difficulties with the institutional church’s teachings on premarital sex prior to the 1960s [...] been underestimated, so too have their tacit but often unvoiced circumventions of traditional teaching on artificial means of contraception’.^17^ Similarly, in the American context, Leslie Tentler has shown through the use of laity testimonials in the 1960s, before the advent of *Humanae Vitae*, that ‘most Catholics came gradually to a full sense of moral autonomy – a process nearly always connected to agonising over contraception’.^18^ Indeed, she argues that most Catholic couples in the United States, ‘seem to have made their peace not only with contraception but independent decision making – if not before the encyclical then shortly after’.^19^

Building on these themes, the recent edited collection by Alana Harris is an important intervention in the history of European men and women’s experiences relating to birth control in the years before and after *Humanae Vitae*. The collection is a pioneer in illustrating how Catholic men and women responded to the papal encyclical in 1968. It also explores the debates surrounding the contraceptive pill, and the ways in which the news media and lay Catholics reacted to the encyclical. It illustrates how these men and women attempted to reconcile their faith with the sexual climate of the 1960s, ‘and their anguished engagement with and interrogation of the papal prohibition’.^20^ Articles in this special issue contribute to these themes too by showing how, in post-contraceptive pill Ireland (Kelly) and twentieth-century Rwanda (Jessee), women navigated through formal and informal contraception bans and, in this process, negotiated gender norms and personal and predominant religious beliefs.

Erin Jessee uses some of the already documented oral traditions (*ibitéekerezo*) as well as new oral testimonies to tackle the relationship between gender norms and practices linked to controlling birth (*kuboneza urubyaro*) in twentieth-century Rwanda. Jessee pays particular attention to the influence of Catholicism, aligned with Belgian colonial rule, on the gendering of family planning in the country, and its contemporary intersections with state-proposed family planning programmes in one of the most densely populated nations in the world. The author explains why, despite widespread support for gender equality and women’s empowerment amongst Rwandans, family planning programmes are viewed with suspicion. Jessee traces the long history of Rwandans’ attitudes towards contraception back to the ‘the Myth of Kigwa’, which exemplifies the enormous value placed on children in Rwanda as well as confinement of reproduction to marriage. She also shows how rigid, but at the same time variable, gender norms – which produced different statuses of women as by-products of their marital and mothering situation and the position of their spouses – influence ideas around family planning and people’s reproductive decisions. The article demonstrates how expansion of Catholicism and colonisation curtailed women’s possibilities of becoming spiritual leaders, severely restricting women’s public presence, and argues that the implementation of twentieth-century public programmes promoting reproductive health and family planning has been crafted as a compromise between the government and the Catholic Church.

## 4 Activists and Experts in Local and Global Settings

Much of the existing histories of birth control activism in the West, particularly in the USA, have emphasised the cross-border circulation of people (such as the emblematic leader Margaret Sanger) and ideas as crucial for the development of the American family planning movement.^21^ This internationalisation became institutionalised in the mid-twentieth century with the advent of the International Planned Parenthood Federation and other organisations, which promoted birth (or population) control across the ‘First’/‘Third’ World political and economic divide.^22^ The ‘Second’ World – central and eastern Europe and the USSR – have been, with a few notable exceptions,^23^ largely excluded from these discussions, a gap Kuźma-Markowska and Ignaciuk’s contribution in this special issue seeks to fill.

Kuźma-Markowka and Ignaciuk’s article focuses on competing, but, as they show, at the same time complementary models of provision of family planning advice in state-socialist Poland during the four decades following decriminalisation of abortion for social reasons in 1956, which the health authorities made available on demand three years later. They demonstrate how different Polish family planning providers, including well-women’s clinics pertaining to public healthcare, state-sponsored but autonomous family planning clinics run by or under auspices of the Society for Conscious Motherhood (SCM; the state-sponsored family planning organisations with close links to the International Planned Parenthood Federation and the British Family Planning Association), and centres run by the Catholic Church used transnational debates, models and organisations for self-legitimisation. The authors also reveal an unexpected cross-fertilisation of ideas and practices between the secular and religious family planning ideologies, and place it in the context of shifting population policy priorities of the communist state, which in the late 1950 clashed but from the 1970s onward became aligned with those of the Catholic Church.

Another important theme in scholarship has been feminist activism linked to contraception – in promoting access, knowledge, informed consent and safety of contraceptive technologies.^24^ Likewise, while many have examined the histories of abortion rights’ and anti-abortion activism,^25^ others have shown examples of religion-based networks and groups which in fact facilitated access to abortion or contraception. This was the case of David P. Cline’s work on Clergy Consultation Service for Abortion, which operated in the USA and Canada in the 1960s, or Raúl Necochea López’s account of the Catholic family planning services in Peru between 1967 and 1976.^26^ In Latin America in particular, the emergence of the liberation theology in the 1960s, with its focus on the embodied experiences of the marginalised, prompted the possibility for a more flexible interpretation of the role of contraception in poor communities.^27^

Ann-Sophie Crosetti’s article in this special issue provides a further example of the unexpected alliances between Catholicism and contraceptive advice through her examination of Belgian Catholic family planning activism in the 1950s and 1960s. Using oral history interviews, personal archives and media accounts, Crosetti traces the trajectories of a dozen secular Catholic counsellors, who worked as volunteer marital advisers in Catholic family planning centres in Brussels, Mons, Namur and Liége. The author places emphasis on the intense secularisation and professionalisation of Catholic marital counselling in Belgium during the 1960s, a process which enabled absorption of international ideas and practices, especially those stemming from the North American humanistic psychology, but also from French and Dutch family planning movements. Crosetti analyses how these influences forged a formation of the counsellors’ professional ethics, who saw their mission as helping clients to negotiate their Catholic identities with both their reproductive choices and Church-imposed institutional norms. These norms became rigidified after almost a decade of discussion within the Catholic world with the 1968 encyclical *Humanae Vitae* which re-validated the ban on ‘artificial’ contraception, particularly the pill. Eventually, the counsellors ended up defending what they called women’s ‘responsible freedom’ in the reproductive realm. The author also examines how the counsellors themselves responded to the encyclical, shedding light on the scarcely examined theme of progressive Catholic activism in the realm of family planning.

In terms of contraceptive expertise, numerous studies have shown the role of doctors, often in interplay with local governments and religious authorities, in the introduction and delivery of family planning services. In Ireland, doctors played an important role in reinforcing Catholic Church teachings and helping to implement the ban on contraception in 1935.^28^ In Spain, as Teresa Ortiz and Agata Ignaciuk have argued, family planning activism was initiated by members of the medical profession before it was popularised by members of radical women’s groups.^29^ Similarly, as Jesse Olszynko-Gryn and Caroline Rusterholz’s recent special issue in this journal has shown, doctors played a crucial role in shaping practice and public opinion in relation to reproductive politics in Britain and France. Mobilising the press in order to disseminate information, they helped to influence policymakers, but also helped to communicate new knowledge to their patients in private settings.^30^ For instance, in England, women doctors such as Helena Wright, Margaret Jackson, Joan Malleson and Gladys Cox, played a significant role in the production and circulation of family planning information there, and to a lesser extent in France, between 1930 and 1970.^31^ Studies of family planning in European contexts, have similarly shown the importance of male doctors in debates around the contraceptive pill.^32^

Contributions to this special issue enrich the understanding of contraceptive expertise and activism by showing examples of more nuanced, entangled forms of involvement in which expertise and activism merge in different political contexts. The socialist state-sponsored Polish SCM is one example of an expert/activist organisation which does not easily fit the interpretational frameworks that align activism with radical, voluntary and autonomous work often critical of the institutions. In the Polish case, the SCM was supported by the communist authorities and served their aims, but at the same time also developed the important, and in many ways radical and pioneering, task of mainstreaming contraceptive advice in a largely Catholic country. On the other hand, Polish Catholic hierarchy and laity’s involvement with anti-contraception family planning counselling in opposition to the Party-state’s policy was also a form of activism, and their self-positioning as experts in reproductive health likewise deserves careful examination.

We see this special issue as an important intervention for three reasons. First, its articles explore the experiences of men and women in three continents and five countries with different political regimes, and interrogate the importance of looking at differing socio-political contexts and the role of churches, doctors and governments, as well as international organisations, in individuals’ personal reproductive choices and practices. Second, we hope that this special issue will be a springboard for future studies of family planning in predominantly Catholic countries from a perspective that pays attention to cross-border flows of knowledge, ideas and practices, as all of the articles in this special issue do. The methodological framework utilised by the contributors draws on a ‘bottom-up’ approach through the use of oral histories, personal archives and media accounts. Finally, it is crucial that we as historians do not look at our individual countries in a vacuum. A transnational approach may help us to fully uncover the commonalities of individual experiences and activist practices across the world.

## Footnotes

1.

Sanjoy Bhattacharya , ‘Global and local histories of medicine: interpretative challenges and future possibilities’, in Mark Jackson (ed.), A Global History of Medicine (Oxford: Oxford University Press, 2018).

2.

Josep L. Barona , ‘Health policies in the twentieth century: a transnational issue’, Health Policies in Interwar Europe: A Transnational Perspective (Oxford, New York: Routledge, 2019), 1–16 (1–2).

3.

Akira Irye and Pierre-Yves Saunier , The Palgrave Dictionary of Transnational History (Basingstoke: Palgrave Macmillan, 2013), XVIII.

4.

Jürgen Osterhammel , ‘A “transnational” history of society continuity or new departure?’, in Heinz-Gerard Haupt and Jürgen Kocka (eds), Comparative and Transnational History: Central European Approaches and New Perspectives (New York, London: Berghahn Books, 2010), 39–51 (46–7).

5.
Caroline Rusterholz, ‘*Deux enfants c’est déjà pas mal’, Famille et fécondité en Suisse, 1955–70* (Lausanne: Editions Antipodes, 2017); Caroline Rusterholz, ‘Reproductive Behaviour and Contraceptive Practices in Comparative Perspective, Switzerland (1955–1970)’, *The History of the Family*, 20, 1 (2015), 41–68; Diane Gervais and Danielle Gauvreau, ‘Women, Priests, and Physicians: Family Limitation in Quebec, 1940–1970’, *Journal of Interdisciplinary History*, 34, 2 (2003), 293–314. For a comparative approach, see Yuliya Hilevych and Caroline Rusterholz, ‘“Two Children to Make Ends Meet”: The Ideal Family Size, Parental Responsibilities and Costs of Children on Two Sides of the Iron Curtain During the Post-War Fertility Decline’, *History of the Family*, 23 3 (2018), 408–25.

6.

*Humanae Vitae and the Damage done: How the Vatican’s Ban on Birth Control Hurt the World* (Catholics for Choice report, 2018).

7.

Lara Marks , Sexual Chemistry: A History of the Contraceptive Pill (New Haven, CT: Yale University Press, 2010).

8.
See for instance: Elizabeth Siegel Watkins, *On the Pill: A Social History of Oral Contraceptives 1950–70* (Baltimore: Johns Hopkins University Press, 1998); Elaine Tyler May, *America and the Pill: A History of the Promise, Peril and Liberation* (New York: Basic Books, 2011).

9.

Hera Cook , The Long Sexual Revolution: English Women, Sex, and Contraception: 1800–1975 (Oxford: Oxford University Press, 2004).

10.
See: Teresa Ortiz-Gómez and Agata Ignaciuk, ‘Pregnancy and Labour Cause more Deaths than Oral Contraceptives’, *Public Understanding of Science*, 24, 6 (2015), 658–71; Agata Ignaciuk, ‘Paradox of the pill: Oral contraceptives in Spain and Poland (1960s–1970s)’, in Ann-Katrin Gembries, Theresia Theuke and Isabel Heinemann (eds), *Children by Choice? Changing Values, Reproduction, and Family Planning in the 20th Century* (Berlin: De Gruyter, 2018), 95–111; Tiago Pires Marques, ‘The politics of Catholic medicine: “The pill” and *Humanae Vitae* in Portugal’, in Harris, *op. cit.* (note 5), 161–86; Ulrike Thoms, ‘The contraceptive pill, the pharmaceutical industry, and changes in the patient–doctor relationship in West Germany’, in Teresa Ortiz-Gómez and María Jesús Santesmases (eds), *Gendered Drugs and Medicine: Historical and Socio-cultural Perspectives* (Farnham: Ashgate, 2014), 153–74; Agata Ignaciuk, Teresa Ortiz-Gómez and Esteban Rodríguez-Ocaña, ‘Doctors, women and the circulation of knowledge of oral contraceptives in Spain, 1960s–1970s’, in Ortiz-Gómez and Santesmases, *op. cit.*, 133–52; Eva-Maria Silies, *Liebe, Lust und Last. Die Pille als weibliche Generationserfährung 1960–1980* (Göttingen: Georg-August-Universität, 2008).

11.
Andrea Tone, ‘Making Room for Rubbers: Gender, Technology, and Birth Control before the Pill’, *History and Technology*, 18 (2010), 51–76; Ilana Löwy, ‘“Sexual Chemistry” before the Pill: Science, Industry and Chemical Contraceptives, 1920–1960’, *British Journal for the History of Science*, 44 (2011), 245–74; Andrea Tone, *Devices and Desires: A History of Contraceptives in America. New York* (New York: Hill and Wang, 2010); Caroline Rusterholz, ‘Testing the Gräfenberg Ring in Interwar Britain: Norman Haire, Helena Wright, and the Debate over Statistical Evidence, Side Effects, and Intra-uterine Contraception’, *Journal of the History of Medicine and Allied Sciences*, 72 (2017), 448–67.10.1093/jhmas/jrx04428973592

12.
See: Chikako Takeshita, *The Global Biopolitics of the IUD: How Science Constructs Contraceptive Users and Women’s Bodies* (Cambridge, MA: MIT Press, 2012); Jesse Olszynko-Gryn, ‘Technologies of contraception and abortion’, in Nick Hopwood, Brenecca Gleming and Lauren Kassel (eds), *Reproduction: From Antiquity to the Present Day* (Cambridge: Cambridge University Press, 2018), 535–51; Sarah Shropshire, ‘What’s a Guy To Do?: Contraceptive Responsibility, Confronting Masculinity, and the History of Vasectomy in Canada’, *Canadian Bulletin of Medical History*, 31, 2 (2014),161–82; Ilana Löwy, ‘Spermicides and their female users after World War II: north and south’, in Ortiz-Gómez and Santesmases, *op. cit.* (note 10), 87–112; Anni Dugdale, ‘Intrauterine Contraceptive Devices, Situated Knowledges, and Making of Women’s Bodies’, *Australian Feminist Studies* 15 (2000), 165–76; Heather Munro Prescott, *The Morning After: A History of Emergency Contraception in the United States* (New Brunswick, NJ: Rutgers University Press, 2011); Dorothy Roberts, *Killing the Black Body: Race, Reproduction, and the Meaning of Liberty* (New York: Pantheon, 1997); Wendy Kline, ‘Bodies of Evidence: Activists, Patients, and the FDA Regulation of Depo-Provera’, *Journal of Women’s History*, 22 (2010), 64–87.10.1353/jowh.2010.058820857592

13.
Ben Mechen, ‘“Closer together”: Durex condoms and contraceptive consumerism in 1970s Britain’, in Jennifer Evans and Ciara Meehan (eds), *Perceptions of Pregnancy from the Seventeenth to the Twentieth Century* (Basingstoke: Palgrave Macmillan, 2016), 213–60; Claire Jones, ‘Under the Covers? Contraceptives, Commerce and the Household in Britain, 1880–1960’, *Social History of Medicine*, 29, 4 (2016), 734–56; Jessica Borge, ‘“Wanting it Both Ways”: The London Rubber Company, the Condom and the Pill, 1915–1970’ (unpublished PhD thesis, Birkbeck University, 2017).

14.

Kate Fisher , Birth Control, Sex and Marriage in Britain 1918–60 (Oxford: Oxford University Press, 2006).

15.

Simon Szreter and Kate Fisher , Sex before the Sexual Revolution: Intimate Life in England 1918–63 (Cambridge: Cambridge University Press, 2010).

16.

Rusterholz, ‘*Deux enfants’, op. cit.* (note 5); Rusterholz, ‘Reproductive Behaviour’, *op. cit.* (note 5); Gervais and Gauvreau, *op. cit.* (note 5).

17.

Alana Harris , Faith in the Family: A Lived Religious History of English Catholicism, 1945–82 (Manchester: Manchester University Press, 2014), 162.

18.

Leslie W. Tentler , Catholics and Contraception: An American History (Ithaca: Cornell University Press, 2008), 231.

19.

*Ibid.*, 267.

20.

Alana Harris (ed.), The Schism of ’68: Catholics, Contraception and Humanae Vitae in Europe, 1945–75 (Basingstoke: Palgrave Macmillan, 2018), 2.

21.
For example, Ellen Chesler. *Woman of Valor: Margaret Sanger and the Birth Control Movement in America* (New York: Simon & Schuster, 2007); Esther Katz, Peter C. Engelman and Cathy Moran Hajo (eds), *The Selected Papers of Margaret Sanger. Volume 4. Round the World for Birth Control, 1920–66* (Chicago: University of Illinois Press, 2016); Carol R. McCann, *Figuring the Population Bomb: Gender and Demography in the Mid-Twentieth Century* (Seattle: University of Washington Press, 2017).

22.
Matthew J. Connelly, *Fatal Misconception: The Struggle to Control World Population* (Cambridge: Harvard University Press, 2009); Parry, Manon. *Broadcasting Birth Control: Mass Media and Family Planning* (New Brunswick, NJ: Rutgers University Press, 2013); Löwy, *op. cit.* (note 11).

23.
For example, Amy E. Randall, ‘“Abortion Will Deprive You of Happiness!”: Soviet Reproductive Politics in the Post-Stalin Era’, *Journal of Women’s History,*23, 3 (2011), 13–38; Yuliya Hilevych and Chizu Sato, ‘Popular Medical Discourses on Birth Control in the Soviet Union during the Cold War: Shifting Responsibilities and Relational Values’, Gembries *et al.*, *op. cit.* (note 9), 99–121; Eszter Varsa, ‘The Gypsy population is constantly growing: Roma and the politics of reproduction in Cold War Hungary’, in Heike Karge, Friederike Kind-Kovacs and Sara Bernasconi (eds), *From the Midwife’s Bag to the Patient’s File: Public Health in Eastern Europe* (Budapest: CEU Press, 2017), 263–91.

24.
Kathy Davis, *The Making of Our Bodies, Ourselves: How Feminism Travels across Borders* (Durham: Duke University Press, 2007); Bibia Pavard, *Si je veux, quand je veux. Contraception et avortement dans la société française (1956–79)* (Rennes: Presses Universitaires de Rennes, 2012); Wendy Kline, *Bodies of Knowledge: Sexuality, Reproduction, and Women’s Health in the Second Wave* (Chicago: University of Chicago Press, 2010); Jennifer Nelson, *Women of Color and the Reproductive Rights Movement* (New York: New York University Press, 2003).

25.
For abortions-rights activism see for instance: Joanna Mishtal and Lorena Anton (eds), *A Fragmented Landscape: Abortion Governance and Protest Logics in Europe* (New York; London: Berghahn, 2017); Gayle Davis and Christabelle Sethna, *Abortion across Borders: Transnational Travel and Access to Abortion Services* (Baltimore: Johns Hopkins University Press, 2019); Kristin Luker, *Abortion and the Politics of Motherhood* (Berkeley: University of California Press, 1984); Sandra McAvoy, ‘From anti-amendment campaigns to demanding reproductive justice: the changing landscape of abortion rights activism in Ireland, 1983–2008’, in J. Schweppe, (ed.), *The Unborn Child, Article 40.3.3 and Abortion in Ireland: Twenty-Five Years of Protection?* (Dublin: Liffey Press, 2008), 39–60; Christaballe Sethna and Steve Hewitt, ‘Clandestine Operations: The Vancouver Women’s Caucus, the Abortion Caravan, and the RCMP’, *Canadian Historical Review*, 90, 3 (2009), 463–95; Faye D. Ginsburg, *Contested Lives: the Abortion Debate in an American Community* (Berkeley: University of California Press, 1989); Faye Ginsburg, ‘Procreation Stories: Reproduction, Nurturance and Procreation in Life Narratives of Abortion Activists’, *American Ethnologist*, 14, 4 (1987), 623–36. On anti-abortion activism, see for example: Karissa Haugeberg, *Women against Abortion: Inside the Largest Moral Reform Movement of the Twentieth Century* (Champaign: University of Illinois Press, 2017); Carol J.C. Maxwell, *Pro-life Activism in America: Meaning, Motivation and Direct Action* (Cambridge: Cambridge University Press, 2002).

26.
Raúl Necochea López, *A History of Family Planning in Twentieth-Century Peru* (Chapel Hill, NC: University of North Carolina Press, 2014), and ‘Priests and Pills: Catholic Family Planning in Peru, 1967–1976’, *Latin American Research Review*, 43, 2 (2008), 34–56; David P. Cline, *Creating Choice: A Community Responds to the Need for Abortion and Birth Control, 1961–73* (New York: Palgrave Macmillan, 2006).

27.

Thia Cooper , ‘Race, Class, and Abortion: How Liberation Theology Enhances the Demand for Reproductive Justice’, Feminist Theology, 24, 3 (2016), 226–244.

28.

Lindsey Earner-Byrne , ‘Moral prescription: the Irish medical profession, the Roman Catholic church and the prohibition of birth control in twentieth-century Ireland’, in Catherine Cox and Maria Luddy (eds), Cultures of Care in Irish Medical History, 1750–1950 (London: Palgrave Macmillan, 2010), 207–228.

29.

Teresa Ortiz-Gómez and Agata Ignaciuk , ‘The Fight for Family Planning in Spain during Late Francoism and the Transition to Democracy, 1965–1979’, Journal of Women’s History, 20, 2 (2018), 38–62.

30.

Jesse Olszynko-Gryn and Caroline Rusterholz , ‘Reproductive Politics in Twentieth-Century France and Britain’, Medical History, 63, 2 (2019), 117–133 (132).3091249710.1017/mdh.2019.1PMC6434651

31.

Caroline Rusterholz , ‘English Women Doctors, Contraception and Family Planning in Transnational Perspective (1930s–70s)’, Medical History, 63, 2 (2019), 153–172.3091249910.1017/mdh.2019.3PMC6434646

32.
Ortiz-Gómez and Ignaciuk, ‘Pregnancy and Labour’, *op. cit.* (note 10).

